# Proceedings of the 11th Annual Deep Brain Stimulation Think Tank: pushing the forefront of neuromodulation with functional network mapping, biomarkers for adaptive DBS, bioethical dilemmas, AI-guided neuromodulation, and translational advancements

**DOI:** 10.3389/fnhum.2024.1320806

**Published:** 2024-02-21

**Authors:** Kara A. Johnson, Nico U. F. Dosenbach, Evan M. Gordon, Cristin G. Welle, Kevin B. Wilkins, Helen M. Bronte-Stewart, Valerie Voon, Takashi Morishita, Yuki Sakai, Amanda R. Merner, Gabriel Lázaro-Muñoz, Theresa Williamson, Andreas Horn, Ro'ee Gilron, Jonathan O'Keeffe, Aryn H. Gittis, Wolf-Julian Neumann, Simon Little, Nicole R. Provenza, Sameer A. Sheth, Alfonso Fasano, Abbey B. Holt-Becker, Robert S. Raike, Lisa Moore, Yagna J. Pathak, David Greene, Sara Marceglia, Lothar Krinke, Huiling Tan, Hagai Bergman, Monika Pötter-Nerger, Bomin Sun, Laura Y. Cabrera, Cameron C. McIntyre, Noam Harel, Helen S. Mayberg, Andrew D. Krystal, Nader Pouratian, Philip A. Starr, Kelly D. Foote, Michael S. Okun, Joshua K. Wong

**Affiliations:** ^1^Norman Fixel Institute for Neurological Diseases, University of Florida, Gainesville, FL, United States; ^2^Department of Neurology, University of Florida, Gainesville, FL, United States; ^3^Department of Neurology, Washington University School of Medicine, St. Louis, MO, United States; ^4^Department of Radiology, Washington University School of Medicine, St. Louis, MO, United States; ^5^Department of Physiology and Biophysics, University of Colorado School of Medicine, Aurora, CO, United States; ^6^Department of Neurosurgery, University of Colorado School of Medicine, Aurora, CO, United States; ^7^Department of Neurology and Neurological Sciences, Stanford University School of Medicine, Stanford, CA, United States; ^8^Department of Psychiatry, University of Cambridge, Cambridge, United Kingdom; ^9^Department of Neurosurgery, Fukuoka University Faculty of Medicine, Fukuoka, Japan; ^10^ATR Brain Information Communication Research Laboratory Group, Kyoto, Japan; ^11^Department of Psychiatry, Graduate School of Medical Science, Kyoto Prefectural University of Medicine, Kyoto, Japan; ^12^Center for Bioethics, Harvard Medical School, Boston, MA, United States; ^13^Department of Psychiatry, Massachusetts General Hospital, Boston, MA, United States; ^14^Department of Neurosurgery, Massachusetts General Hospital, Boston, MA, United States; ^15^Department of Neurology, Center for Brain Circuit Therapeutics, Harvard Medical School, Brigham & Women's Hospital, Boston, MA, United States; ^16^MGH Neurosurgery and Center for Neurotechnology and Neurorecovery (CNTR) at MGH Neurology Massachusetts General Hospital, Harvard Medical School, Boston, MA, United States; ^17^Movement Disorder and Neuromodulation Unit, Department of Neurology, Charité – Universitätsmedizin Berlin, Corporate Member of Freie Universität Berlin and Humboldt- Universität zu Berlin, Berlin, Germany; ^18^Rune Labs, San Francisco, CA, United States; ^19^Machine Medicine, London, United Kingdom; ^20^Biological Sciences and Center for Neural Basis of Cognition, Carnegie Mellon University, Pittsburgh, PA, United States; ^21^Department of Neurological Surgery, Weill Institute for Neurosciences, University of California, San Francisco, San Francisco, CA, United States; ^22^Department of Neurosurgery, Baylor College of Medicine, Houston, TX, United States; ^23^Edmond J. Safra Program in Parkinson's Disease, Division of Neurology, Morton and Gloria Shulman Movement Disorders Clinic, Toronto Western Hospital, University Health Network (UHN), University of Toronto, Toronto, ON, Canada; ^24^Krembil Brain Institute, Toronto, ON, Canada; ^25^Restorative Therapies Group Implantables, Research, and Core Technology, Medtronic Inc., Minneapolis, MN, United States; ^26^Boston Scientific Neuromodulation Corporation, Valencia, CA, United States; ^27^Neuromodulation Division, Abbott, Plano, TX, United States; ^28^NeuroPace, Inc., Mountain View, CA, United States; ^29^Department of Engineering and Architecture, University of Trieste, Trieste, Italy; ^30^Newronika SPA, Milan, Italy; ^31^Department of Neuroscience, West Virginia University, Morgantown, WV, United States; ^32^Medical Research Council Brain Network Dynamics Unit, Nuffield Department of Clinical Neurosciences, University of Oxford, Oxford, United Kingdom; ^33^Edmond and Lily Safar Center (ELSC) for Brain Research and Department of Medical Neurobiology (Physiology), Institute of Medical Research Israel-Canada, Hebrew University of Jerusalem, Jerusalem, Israel; ^34^Department of Neurosurgery, Hadassah Medical Center, Jerusalem, Israel; ^35^Department of Neurology, University Medical Center Hamburg-Eppendorf, Hamburg, Germany; ^36^Department of Neurosurgery, Center for Functional Neurosurgery, Ruijin Hospital, Shanghai Jiao Tong University School of Medicine, Shanghai, China; ^37^Neuroethics, Department of Engineering Science and Mechanics, Philosophy, and Bioethics, and the Rock Ethics Institute, Pennsylvania State University, State College, PA, United States; ^38^Department of Biomedical Engineering, Duke University, Durham, NC, United States; ^39^Department of Neurosurgery, Duke University, Durham, NC, United States; ^40^Department of Radiology, Center for Magnetic Resonance Research, University of Minnesota, Minneapolis, MN, United States; ^41^Department of Neurology, Neurosurgery, Psychiatry, and Neuroscience, Icahn School of Medicine at Mount Sinai, New York, NY, United States; ^42^Departments of Psychiatry and Behavioral Science and Neurology, University of California, San Francisco, San Francisco, CA, United States; ^43^Department of Neurological Surgery, University of Texas Southwestern Medical Center, Dallas, TX, United States; ^44^Department of Neurosurgery, University of Florida, Gainesville, FL, United States

**Keywords:** deep brain stimulation (DBS), artificial intelligence, neuroethics, interventional psychiatry, adaptive DBS, Parkinson's disease, epilepsy, optogenetics

## Abstract

The Deep Brain Stimulation (DBS) Think Tank XI was held on August 9–11, 2023 in Gainesville, Florida with the theme of “Pushing the Forefront of Neuromodulation”. The keynote speaker was Dr. Nico Dosenbach from Washington University in St. Louis, Missouri. He presented his research recently published in Nature inn a collaboration with Dr. Evan Gordon to identify and characterize the somato-cognitive action network (SCAN), which has redefined the motor homunculus and has led to new hypotheses about the integrative networks underpinning therapeutic DBS. The DBS Think Tank was founded in 2012 and provides an open platform where clinicians, engineers, and researchers (from industry and academia) can freely discuss current and emerging DBS technologies, as well as logistical and ethical issues facing the field. The group estimated that globally more than 263,000 DBS devices have been implanted for neurological and neuropsychiatric disorders. This year's meeting was focused on advances in the following areas: cutting-edge translational neuromodulation, cutting-edge physiology, advances in neuromodulation from Europe and Asia, neuroethical dilemmas, artificial intelligence and computational modeling, time scales in DBS for mood disorders, and advances in future neuromodulation devices.

## Introduction

The 11th Annual Deep Brain Stimulation (DBS) Think Tank meeting was held on August 9–11, 2023 at the University of Florida in Gainesville, Florida and virtually for those not attending in-person (Zoom Video Communications). There have now been an estimated 263,000 DBS devices implanted for neurological and neuropsychiatric disorders worldwide. The DBS Think Tank was founded in 2012 and provides an open platform where clinicians, engineers, and researchers (from industry and academia) can freely discuss current and emerging DBS technologies, as well as logistical and ethical issues facing the field. The DBS Think Tank emphasizes cutting-edge research and collaboration, aiming to more rapidly advance the neuromodulation field.

The DBS Think Tank meeting was focused on advances in the following areas:

1. Cutting-edge translational neuromodulation, including optogenetics and vagus nerve stimulation;
2. Neurophysiology to guide adaptive DBS (aDBS) and to identify neural biomarkers of mood;
3. DBS research in Europe and Asia focused on neurophysiology, sleep, gait, and neuropsychiatry (depression and Tourette syndrome);
4. Neuroethical dilemmas that will likely inform the future of neuromodulation;
5. Cutting-edge artificial intelligence (AI) and computational modeling;
6. Time scales to measure and treat mood disorders with DBS;
7. Critical features to be incorporated in future DBS devices.

These proceedings will summarize the sessions and discussions held at the 11th Annual DBS Think Tank meeting.

## The somato-cognitive action network (scan) as a target for neuromodulation

**Figure 1:**
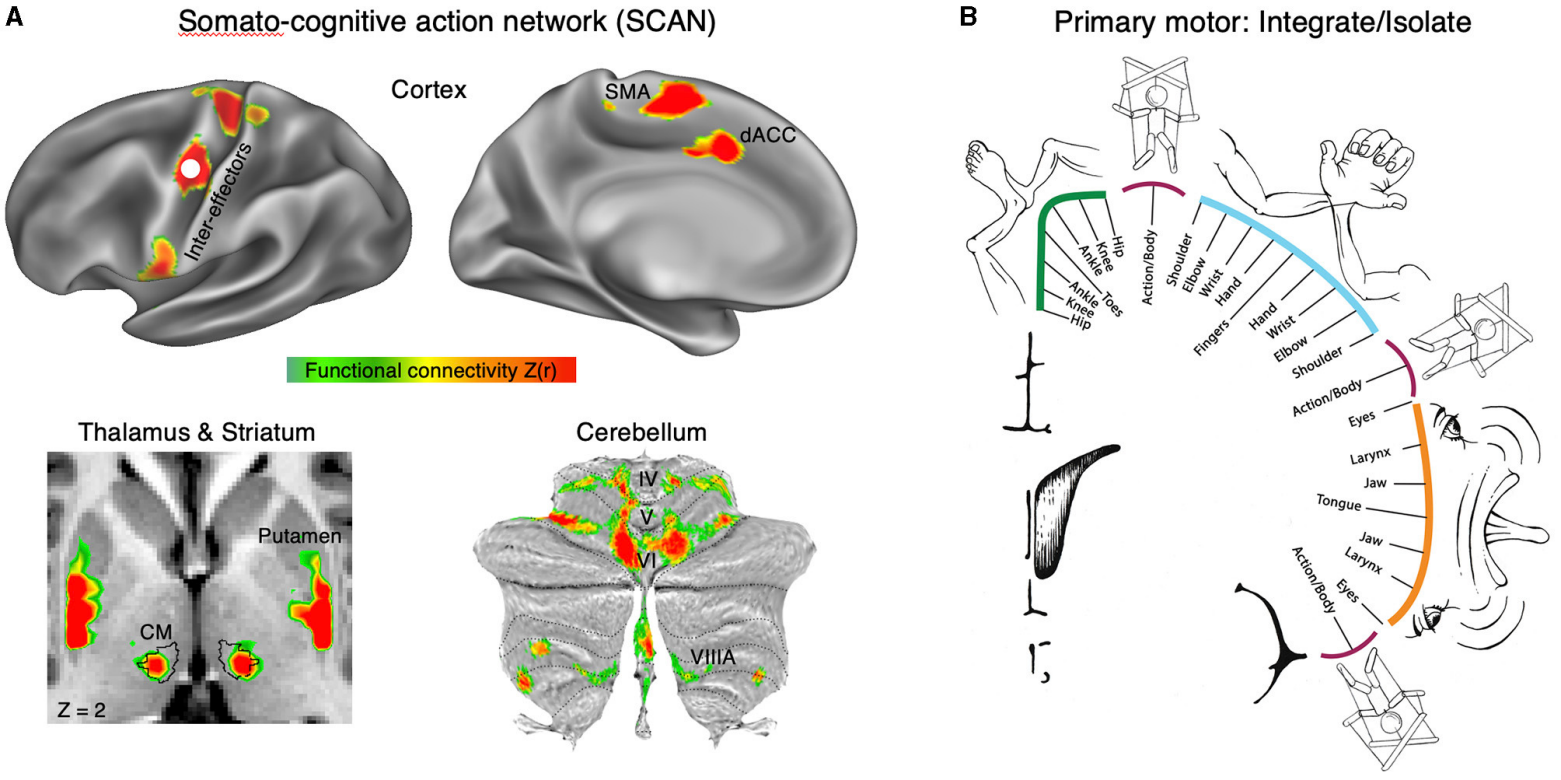
Somato-cognitive action network (SCAN). **(A)** Resting state functional connectivity (RSFC) seeded from the middle inter-effector node in primary motor cortex (bilaterally) in a representative individual (P1; 356 min resting-state fMRI). In cortex the SCAN includes three inter-effector nodes (superior, middle, inferior) that alternate with effector-specific foot, hand and mouth primary motor regions, as well as two nodes on the dorsal midline in the SMA (supplementary motor area) and dACC (dorsal anterior cingulate cortex) that are interleaved with the effector-specific regions of the SMA/pre-SMA. In thalamus, the centromedian nucleus [CM; black outline (Ewert et al., 2017)] is part of the SCAN. In the striatum, the dorsal posterior putamen forms part of the SCAN. In the cerebellum, crus VI and para-vermian VIIIA are part of the SCAN. Functional connectivity [Z(r)] in cortex was thresholded from 0.35 to 0.6, and from 0.15 to 0.3 to account for the lower signal-to-noise ratio. **(B)** In the integrate–isolate model of M1 organization, effector-specific—foot (green), hand (cyan) and mouth (orange)—functional zones are represented by concentric rings with proximal body parts surrounding the relatively more isolatable distal ones (toes, fingers and tongue). The SCAN inter-effector regions (maroon) sit at the intersecting points of these fields and support integrative, allostatic whole-body control.

Dr. Nico Dosenbach was the invited keynote speaker, who presented on the story behind his research identifying the somato-cognitive action network (SCAN) together with Dr. Evan Gordon and their collaborative team. They discovered that two parallel systems intertwine in the brain's motor circuits forming an integrate–isolate pattern: effector-specific regions (foot, hand and mouth) for isolating fine motor control and the somato-cognitive action network (SCAN) for integrating goals, physiology, and body movement (Figure 1) (Gordon et al., 2023; Graziano, 2023; Leopold, 2023).

The SCAN regions are strongly functionally connected to each other and to the cingulo-opercular network (CON), which is critical for action (Dosenbach et al., 2006) and physiological control (Pool and Ransohoff, 1949), arousal (Wall and Davis, 1951), errors (Neta et al., 2015), and pain (Hoeppli et al., 2022). In primary motor cortex, concentric effector somatotopies are interrupted by SCAN inter-effector regions. The SCAN inter-effectors lack movement specificity and co-activate during action planning (coordination of hands and feet) and axial body movement (such as of the abdomen or eyebrows).

The SCAN may be directly relevant to advancing neuromodulation. The SCAN includes two thalamic DBS targets: the ventral intermediate (VIM) nucleus for tremor (Ondo et al., 1998; Fasano et al., 2012) and the centromedian (CM) nucleus for epilepsy (Valentín et al., 2013) and Tourette syndrome (Schrock et al., 2015). The anti-tremor effects of the VIM may be mediated by its connectivity to the cerebellum, and the CM's role in arousal may explain its anti-epileptic effects. Additionally, Parkinson's disease (PD) may be related to dysfunction of SCAN circuitry, as PD symptoms cut across motor, physiological and volitional domains [e.g., postural instability, autonomic dysfunction, and reduced self-initiated activity (Dauer and Przedborski, 2003; Bloem et al., 2021)]. Therefore, the SCAN may partially explain the therapeutic effects of DBS in disorders that involve complex motor and nonmotor integration, which could inform novel patient-specific functional mapping for targeted neuromodulation.

## Cutting edge translational neuromodulation

Over the past decade, there has been an influx of bench research focused on understanding the therapeutic mechanisms of neuromodulation (Wong et al., 2023). Optogenetics has provided key insights into cell-type specific effects of DBS and developing novel stimulation paradigms for more selective stimulation. Additionally, there is increased interest in understanding the role of learning and neuroplasticity in specific brain circuits in driving motor and nonmotor effects of neuromodulation.

### Circuit-inspired strategies to improve treatments for Parkinson's disease

The identification of distinct cell-types throughout the basal ganglia has been essential in advancing the understanding of network function and improving neurological therapies. In the globus pallidus externa (GPe), interventions targeting neuronal subpopulations have profound therapeutic potential, but are challenging to implement in clinical settings. The lab of Dr. Aryn Gittis investigated whether electrical stimulation can be tuned to engage cell-type specific responses in the GPe. Although conventional stimulation was non-specific, brief and high frequency bursts of stimulation elicited bimodal responses of Parvalbumin (PV-GPe) and Lim homeobox 6 (Lhx6-GPe) subpopulations. In dopamine depleted mice, burst-DBS stimulation optimized for cell-type specificity induced motor recovery with sustained therapeutic benefits that persisted for hours after the offset of stimulation. These results establish the feasibility of shaping electrical stimulation patterns to drive population-specific neuromodulation in the central nervous system and suggest a potential for developing a more robust toolbox for DBS therapies in humans.

### Modulating neural reinforcement with subthalamic DBS in Parkinson's disease

aDBS provides an unprecedented temporal precision that holds promise to counteract the negative effects of dopamine loss in PD (Neumann et al., 2023a). To achieve this goal, it is crucial to understand the neural circuit dynamics, their function, and their modulation that underlie physiological dopamine signaling. It was recently proposed that the shared effect of dopaminergic signals across limbic, associative, and motor circuits can be neural reinforcement (Athalye et al., 2020), the modulation of strength of neural population dynamics, and their likelihood to reoccur. Translating this concept to PD can spark new hypotheses on the pathogenesis of PD symptoms (Cheung et al., 2023) and ways to counteract them. Given that DBS, like dopamine release, can suppress indirect pathway activity (Neumann et al., 2023b), temporally precise stimulation could mimic the circuit effects of dopamine transients. Early findings from Dr. Julian Neumann's lab support this claim, as kinematic closed-loop DBS shows specific invigorating effects on the behavior at the time during which stimulation is applied. For PD, this could serve as a foundation for neural circuit prosthetics that may reinstate healthy basal ganglia function to counteract pathological reinforcement and support neural learning. If true, this could extend DBS for the adaptation to neuroprosthetics for stroke rehabilitation, spinal cord injury, or retina implants by supporting neural learning.

### Vagus nerve stimulation to enhance motor learning and myelin repair

Closed-loop VNS was recently approved by the FDA to restore upper limb mobility for patients with stroke. Preclinical and early clinical studies suggest that closed-loop VNS improves recovery from conditions such as spinal cord injury, traumatic brain injury, post-traumatic stress disorder, and addiction. Despite the wide-ranging etiology of these conditions, the therapeutic model is similar; VNS is paired with a relevant rehabilitation protocol. The precise timing of stimulation is a key element to drive specific circuit plasticity and functional recovery. Yet, VNS activates widespread brain networks, raising the question of how closed-loop VNS may lead to circuit-specific alterations in plasticity and functional recovery.

**Figure 2:**
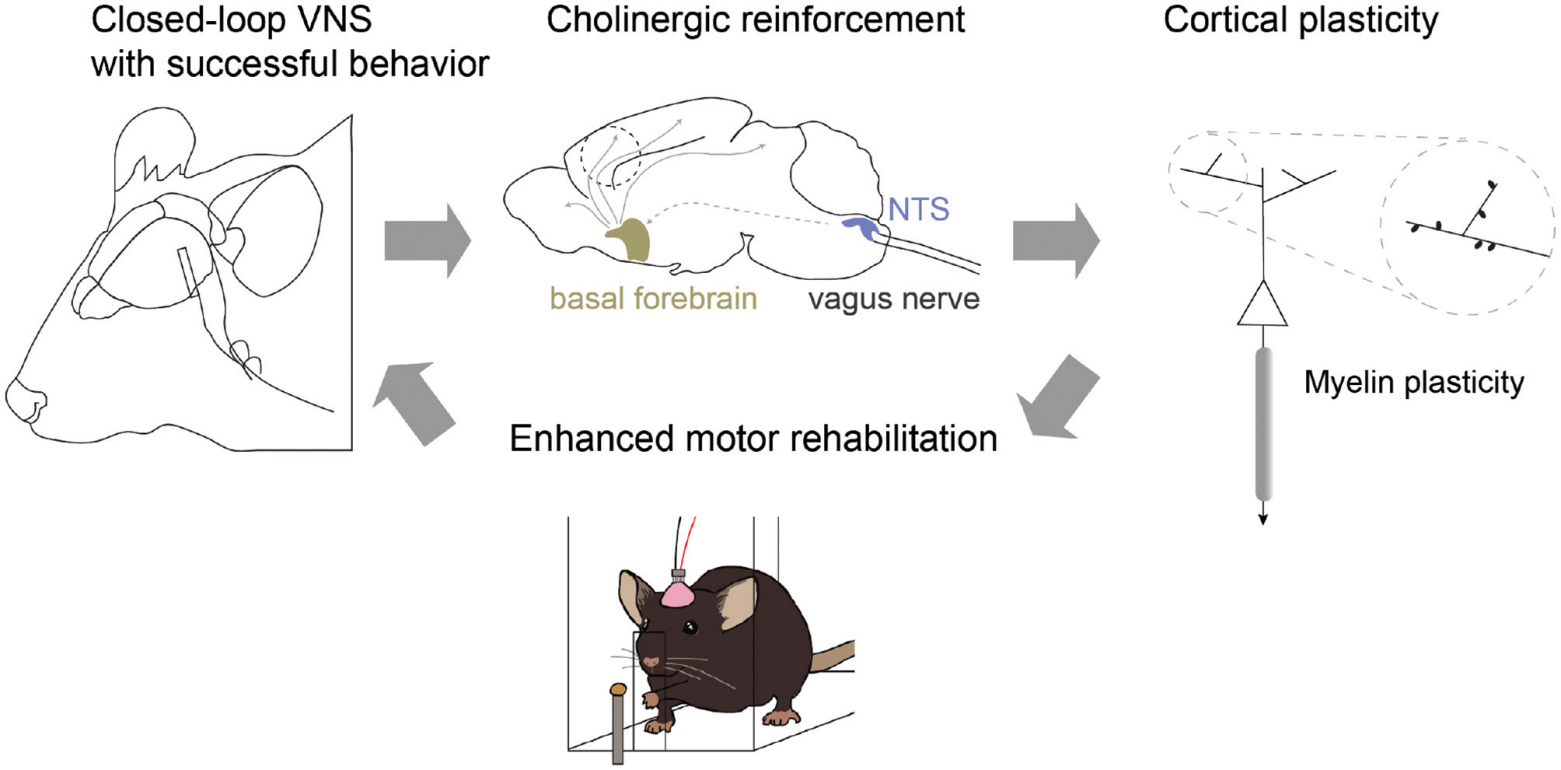
Closed-loop vagus nerve stimulation (VNS) for reinforcement learning. VNS paired with successful behavioral outcome drives phasic cholinergic signaling from the basal forebrain and modulates neuronal representation of outcome within motor cortex. In addition, closed-loop VNS influences myelin plasticity and repair within motor cortex, restoring motor function in models of demyelination. Together, these results suggest a model in which closed-loop VNS can enhance behavioral reinforcement cues through cholinergic neuronal activity to promote circuit-specific plasticity and enhanced learning.

Research from Dr. Cristin Welle's lab has recently found that VNS can enhance motor learning in healthy animals through cholinergic-mediated reinforcement learning (Bowles et al., 2022) (Figure 2). VNS elicits brief cholinergic signaling from the basal forebrain (BF) and brief inhibition of cholinergic neurons during VNS prevents enhanced motor learning. The timing of these signals is critical, as VNS paired with a successful reach improves learning, but VNS paired with the initiation of movement does not. Early results also show evidence that VNS can increase myelination of neural circuits, with specificity of sheath placement increased during VNS paired with success. Together, these results indicate that VNS augments cholinergic reinforcement to enhance learning and plasticity in motor systems.

## Cutting edge physiology in pursuit of adaptive DBS

An ongoing exciting development in the field is aDBS, a paradigm that uses brain activity or wearable sensors to deliver stimulation when symptoms are present. By only delivering intermittent stimulation, aDBS may improve therapeutic efficacy, reduce side effects, and prolong DBS device battery life. However, challenges remain in determining the optimal approach to design aDBS algorithms, including which control signal(s) to use, how to determine patient-specific biomarkers, and how to account for changes in brain state (e.g., during sleep and across circadian cycles).

### Toward automated, data-driven, adaptive DBS

aDBS has great potential to improve therapeutic efficacy, however significant scalability challenges remain due to complexities of implementation. To this end, Dr. Simon Little's lab introduced three proof-of-principle adaptive algorithms toward personalized, automated programming. First, they demonstrated an automated machine learning pipeline for biomarker selection in three patients with PD. Multiple methods converged to show that cortico-basal finely tuned gamma (FTG) outperformed classical subcortical beta as an optimal biomarker. aDBS parameterized against either subthalamic (STN) or cortical FTG showed a significant increase in ON times and improved quality of life (Oehrn et al., 2023). Second, they introduced a new, fully data-driven algorithm in which all parameters—from biomarker identification to aDBS optimization—were parameterized in an automated manner, fully remotely, in patients' own homes. This demonstrated a reduction in dyskinesia and improved movement speeds in a natural typing task. Finally, they demonstrated multi-night at-home sleep recordings and negative interactions between beta and slow waves, in addition to a fully data-driven aDBS algorithm for sleep stage modification targeting NREM sleep (Anjum et al., 2023; Smyth et al., 2023). Their work shows that personalized nighttime stimulation adjustments were well tolerated with initial evidence that this technique might be able to optimize NREM slow waves linked to disease progression.

### Neural biomarkers of mood: implications for the closed-loop debate

DBS of the subcallosal cingulate (SCC) and ventral capsule/ventral striatum (VC/VS) has been used for treatment-resistant depression (TRD) with variable success. Improving our fundamental understanding of the neurophysiological basis of mood state variation would allow for a more data-driven approach to optimizing stimulation delivery, which in turn would likely improve the consistency and predictability of outcomes (Allawala et al., 2021). Dr. Sameer Sheth's lab conducted intracranial recordings in depression-relevant regions in a cohort of three patients with severe depression that were enrolled in an early feasibility trial (NCT03437928) of individualized DBS guided by intracranial recordings (UH3 NS103549). The outcomes of the first subject in the trial were recently reported (Sheth et al., 2022). Each subject was implanted with two pairs of permanent DBS leads for stimulation delivery in the SCC and VC/VS, along with temporary stereo-electroencephalography electrodes for neural recordings. After surgery, subjects underwent inpatient monitoring for 9 days, during which they obtained frequent self-report measures of depression severity and simultaneous network-wide neural recordings. The results indicated that decreased low-frequency and increased high frequency neural activity across prefrontal regions correlated with reduced depression severity. Based on these observations, they built a model to predict depression severity from neural activity and found that high frequency (beta, gamma) power in the dorsal anterior cingulate cortex significantly predicted depression severity across all three subjects. When the model was not constrained to features from a single region, individual-specific sets of spatiospectral features predictive of symptom severity emerged that reflected the heterogeneity of the disorder (Xiao et al., 2023). The ability to predict depression severity based on neural activity increases our understanding of the neural basis of depression and provides a target neural state for personalized stimulation interventions.

### Sensing motion: real time kinematic feedforward and feedback inputs for aDBS

**Figure 3:**
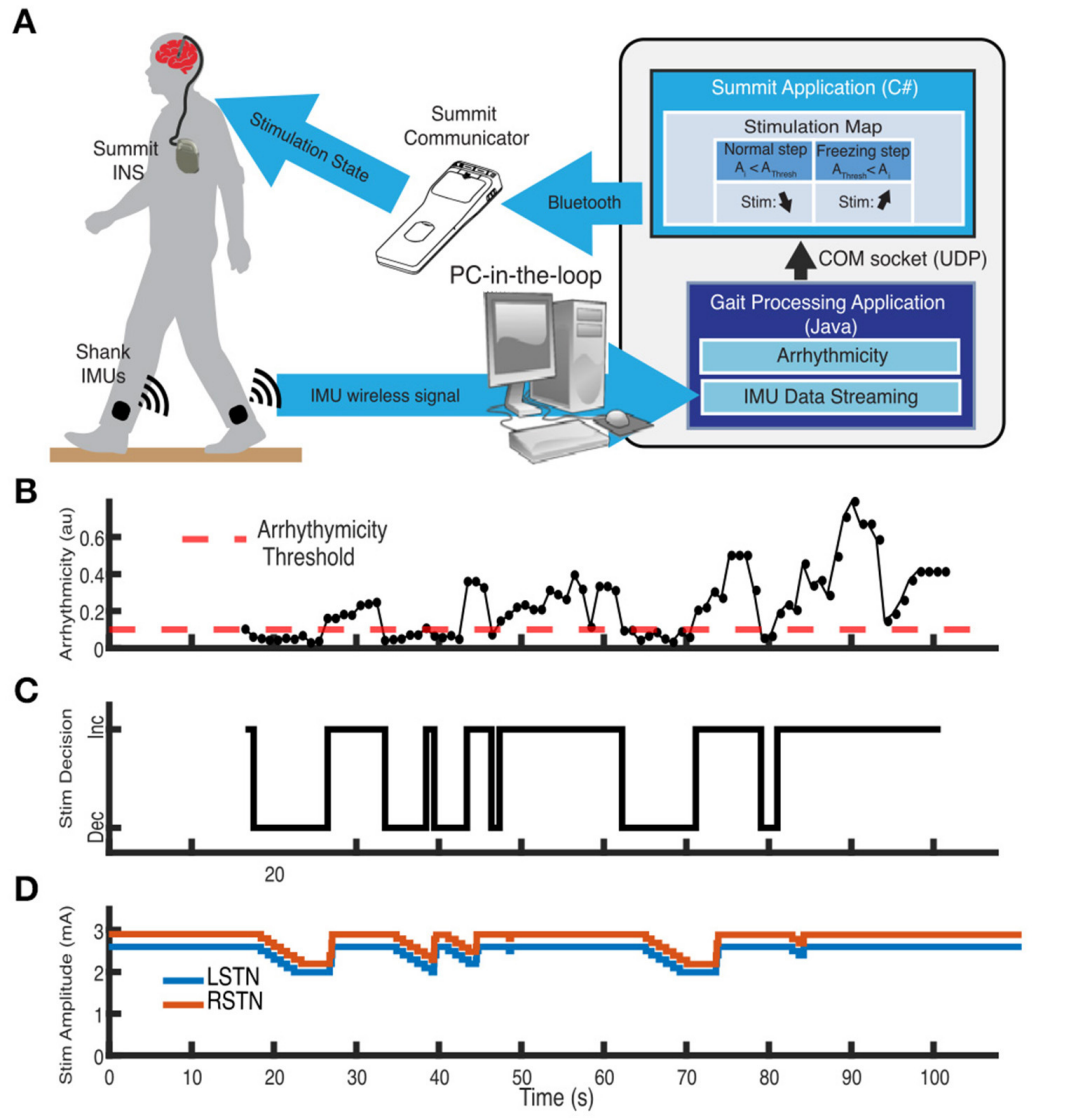
Example of adaptive DBS system for freezing of gait in Parkinson's disease using kinematic inputs. **(A)** Schematic of distributed aDBS system. Inertial measurement unit (IMU) data is streamed in real-time to a PC-in-the-loop. Gait arrhythmicity is measured from the IMU data. A decision to increase or decrease stimulation intensity is made depending on whether the measured arrhythmicity is above or below an established threshold, which is then communicated back to the Summit RC+S neurostimulator via the Summit communicator. **(B)** Measurement of the real-time arrhythmicity over the course of a stepping-in-place task relative to the threshold. **(C)** Stimulation decisions to increase or decrease stimulation based on the arrhythmicity. **(D)** Adaption of stimulation amplitude of the two subthalamic nuclei in response to the gait arrhythmicity. Adapted from Melbourne et al. (2023).

Closed-loop or aDBS relies on inputs that are relevant to neurological symptoms. The primary focus to date has been on using relevant neural signals as inputs for aDBS, often from the site of the DBS lead. However, kinematic inputs derived from wearable sensors on the body offer an opportunity for directly measuring a behavior or symptom of interest with high fidelity. There is growing interest in using kinematic data to classify behaviors. Symptoms such as tremor and freezing of gait can be reliably measured using inertial measurement units placed on various parts of the body. These data can then be used to adapt stimulation to provide therapy in a demand-based fashion. Initial studies have demonstrated the successful implementation and tolerability of an aDBS system based on real-time kinematic inputs for both tremor, across both Parkinson's disease and essential tremor, as well as freezing of gait. These approaches offer the advantages of: not requiring sense-friendly stimulation configurations, being resistant to stimulation-related neural artifact, having a much higher fidelity signal in comparison to subcortical neural signals recorded at the site of stimulation, enabling the use of rapid ramp rates to provide changes in therapy, and allowing adaptation of other stimulation parameters (e.g., frequency). Measuring the kinematics of the symptom targeted for therapy at different DBS intensities facilitates the choice of a therapeutic range that aDBS will be constrained to, thus enabling precise, safe, and tolerable aDBS, driven by relevant kinematics (Figure 3).

## Advances in commercially available neuromodulation technologies

Collaboration between academia and industry is central to progressing the field of DBS. The DBS Think Tank features an industry blitz each year to highlight novel therapies and discuss challenges and opportunities in translating new technologies into products for clinical use.

### Advances in brain sensing

Technological advances in implantable neural stimulators have made it possible to sense brain signals from DBS leads while delivering stimulation. Sensing technology has created unique insight into patient-specific biomarkers and brain states not only when patients are in the clinic but also in the home setting. Commercially approved devices are now on the market with embedded brain sensing technology making chronic brain data during routine clinical care accessible worldwide (Chen et al., 2019; Elder et al., 2019; Arlotti et al., 2021; Goyal et al., 2021). The Medtronic Percept™ device with BrainSense™ technology has now been implanted in tens of thousands of patients globally including for the approved indications of Parkinson's disease (PD), essential tremor, dystonia, epilepsy, dystonia, and obsessive-compulsive disorder (OCD).

Evidence is building that BrainSense may be used to guide DBS programming setup and optimization in movements disorders and epilepsy (Feldmann et al., 2021; Buijink et al., 2022; Fasano et al., 2022; Strelow et al., 2022; Chua et al., 2023; Swinnen et al., 2023). Recent studies in Parkinson's disease suggest BrainSense can be used to streamline and reduce the time for initial DBS programming (Binder et al., 2023; Lewis et al., 2023), troubleshoot difficult cases, identify medication interactions, and mitigate potential overstimulation (Kern et al., 2022; Vaou et al., 2023). Simplified data visualizations are also being developed to expand access to the insights learned to date (Thompson et al., 2023). Collectively, these case studies demonstrate the value of chronic brain signals as objective feedback for simplifying and tailoring DBS therapy management.

Additional research is exploring how BrainSense can be used for aDBS in PD, where an investigational Percept software unlock allows automated stimulation amplitude modulation. The Medtronic-sponsored ADAPT PD trial is evaluating the safety and effectiveness of aDBS (Herrington et al., 2023), whereas several feasibility studies are reporting potential benefits of aDBS over continuous DBS (Nakajima et al., 2021; Oehrn et al., 2023; Smyth et al., 2023). Furthermore, primarily through the NIH BRAIN Initiative other investigations are focusing on developing BrainSense biomarkers and classifiers of neuropsychiatric disorders including treatment-resistant depression, OCD and Tourette syndrome (Vissani et al., 2022; Alagapan et al., 2023; Butson et al., 2023).

In general, as DBS technology continues to advance it will be critical for product designers to consider the balance between routine but clinically meaningful innovation and foundational research. Therefore, Percept is an example platform that has been intentionally designed to be software and firmware upgradable for unlocking both ease of use features as well as advance technical capabilities.

### Toward automated image- and outcomes-guided DBS

Boston Scientific Neuromodulation (BSN) focuses on developing powerful, clinically impactful technology to enable precision control of novel stimulation patterns in the time domain, and automation of stimulation targeting, using image- and outcomes-guided programming strategies.

The Chronos research software unlocks pulse-by-pulse stimulation composition in chronically implanted patients allowing researchers to explore novel stimulation patterns with the goal of improving therapy for current and future indications and expanding understanding of DBS.

Image-guided programming tools GUIDE XT and STIMVIEW XT, provided through a collaboration with Brainlab Inc., allow clinicians to visualize the lead and the stimulation within the patient's own anatomy during surgical planning and patient programming. By correlating anatomy and outcomes from individual stimulation locations across multiple subjects, BSN, in collaboration with multiple centers, is generating probabilistic maps to define “hot” and “sour” spots for stimulation. DBS Illumina 3D then allows clinicians to specify predefined target and avoidance regions and instantly recommends a set of stimulation parameters. BSN is working to make this algorithm commercially available in the future.

Outcomes-guided programming tools, such as Clover, allow the clinicians to further refine initial programming solutions. Clover assists clinicians programming linear (Sasaki et al., 2021) or directional (Wenzel et al., 2021) leads while evaluating multiple symptoms (Gülke et al., 2022). The algorithm is incorporated in the StimSearch™ software and uses physician provided symptom and side effect scores to generate suggested stimulation settings to explore the search space. StimSearch™ interfaces with the BSN clinician programmer and can be made available upon request.

### Advances in digital health integration

Advances in hardware platforms, mobile computing, and sensor technology (with features like bluetooth low energy and upgradable software), along with a favorable regulatory and reimbursement landscape, have resulted in greater digital health integration with device-aided therapies like neuromodulation. Abbott presented a digital health vision for this field by introducing the Neurosphere™ Virtual Clinic, a telehealth platform that helps connect patients with their clinicians so their therapy can be evaluated and adjusted remotely. Digital health integration may expand access, improve outcomes, and drive efficiencies to better serve patients. These technologies may be crucial as DBS expands to other indications, for example, treatment resistant depression, where issues like access, compliance with therapy, and follow-up care are critical because the patient population may be vulnerable. Leveraging ubiquitous consumer-grade technology enables DBS devices to provide tailored solutions through already familiar interfaces and fit better into patient lives.

### Updates in neuromodulation for epilepsy

NeuroPace has ongoing clinical trials studying neuromodulation in epilepsy. The NAUTILUS Study (NCT05147571) is a pivotal study to determine if the RNS System is safe and effective as an adjunctive therapy for the treatment of primary generalized seizures in individuals 12 years and older who have drug-resistant idiopathic generalized epilepsy (IGE). This prospective, multicenter, single-blind, randomized, sham stimulation controlled study will enroll up to 100 participants within the United States. Leads are placed bilaterally in the centromedian nuclei. Primary outcome measures are the 12-week post-operative serious device-related adverse event rate and the time to second generalized tonic-clonic seizure.

The RNS System Lennox-Gastaut Syndrome (LGS) Feasibility Study (NCT05339126) is an NINDS funded Brain Initiative study intended to generate preliminary safety and effectiveness data for brain-responsive neurostimulation of thalamocortical networks as an adjunctive therapy in reducing the frequency of generalized seizures in patients 12 years or older with LGS who are refractory to antiseizure medications. The study is ongoing and up to 20 subjects will be implanted. Leads are placed bilaterally in the prefrontal cortex and centromedian nuclei.

The Patient nSight Report has been updated with expanded patient-specific content to help physicians more efficiently review patient data prior to clinic visits.

An updated version of the Patient Data Management System interface has been released that includes *Simple Set*. This expanded feature allows a complete patient-specific programming set to be selected and sent to the RNS Tablet to streamline physicians' in-clinic experience.

### Passive monitoring to guide Parkinson's disease treatment

**Figure 4:**
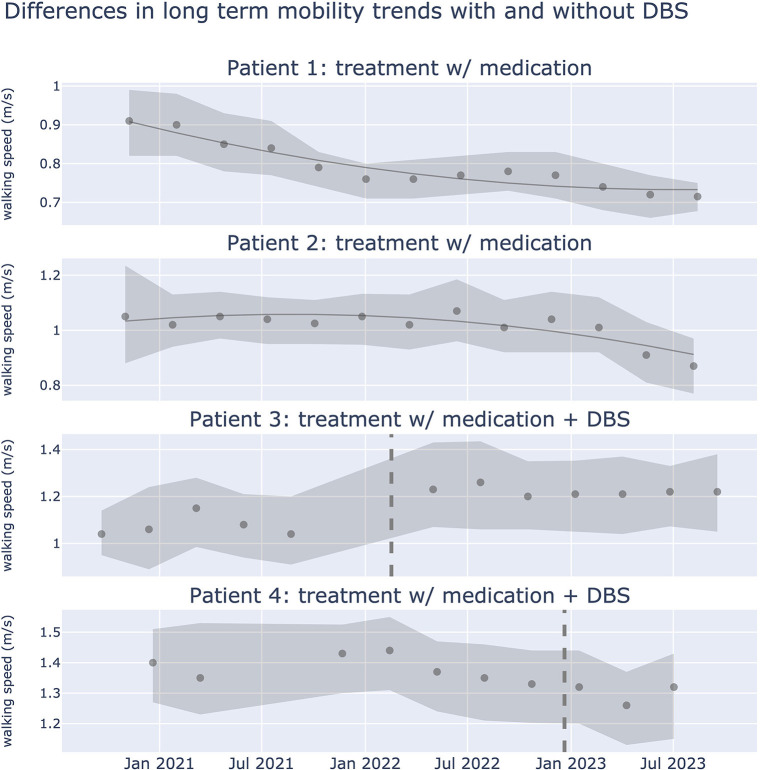
Differences in long term walking speed trends with and without DBS treatment. Gait dysfunction in PD is associated with the cholinergic system, and may be a more sensitive biomarker than other motor signs of PD that respond to medication or DBS. Plots show walking speed over a period of 2.5 years from 4 patients. Walking speed (meters/second) was captured using iPhone actigraphy during real world activities. Scatter points represent median walking speed during a 3 month window, shaded error bars represent the 25th and 75th percentile of walking speed respectively. Dotted line represents the date in which DBS was implanted. Data from 32,550 walking events, across 321.17 walking hours.

Evaluating DBS motor outcomes for Parkinson's disease is challenging due to treatment and patient heterogeneity. While clinical scales such as the Movement Disorders Society (MDS) Unified Parkinson's Disease Rating Scale (UPDRS)-III are the gold standard, they provide intermittent, subjective snapshots during clinical visits. In contrast, passive monitoring of patient symptoms offers continuous, objective monitoring of motor fluctuations in Parkinson's disease in real-life scenarios (Evers et al., 2019; Omberg et al., 2022; Klein and Bloem, 2023). Rune Labs assessed the utility of passive wearable monitoring combined with population level monitoring in (1) predicting short-term DBS effectiveness, and (2) characterizing long-term symptom trajectories. Using shorter time scales (days) they built a model to predict DBS effectiveness in suppressing tremor. Local field potential (LFP) fluctuations and sensor locations were the most important features identified. This model could provide the basis for sensing-guided programming targeted to specific symptom reduction. Over longer time scales, assessing DBS effects becomes challenging due to medication and stimulation changes; they focused on gait metrics less influenced by levodopa or DBS, associated with cholinergic system deficits (Figure 4). They show that such markers may be ideal in capturing progressive progression of Parkinson's disease.

### AlphaDBS for closed-loop DBS

The AlphaDBS System is a local field potential (LFP) sensing enabled, CE-certified, fully implantable, DBS system for the treatment of Parkinson's disease. The system records LFP power in a patient-specific selected band continuously, and this sensing data is provided to the clinician via a cloud-based data management system. The system can operate in both a conventional and aDBS (cDBS-aDBS) mode. In aDBS LFP beta power is used as the input variable to the adaptive proportional control algorithm that computes a new stimulation amplitude every minute (Arlotti et al., 2021).

To date, 11 patients requiring a DBS implantable pulse generator exchange and five new patients have been implanted in a European feasibility study comparing aDBS vs cDBS (ClinicalTrials.gov ID NCT04681534). When, at the end of the study period, patients were asked to blindly choose between the two stimulation modes received, 90% of them preferred aDBS over cDBS.

Data available so far in the cloud-based data management system consist of more than 40,000 h of LFP recordings, with two patients receiving aDBS for more than 1 year and 3 for more than 6 months after the end of the study period. Preliminary analyses show the ability of recorded LFPs to track daily activities, particularly sleep, and to monitor motor behaviors.

## Advances in Europe

### Effect of high frequency STN DBS: beyond beta suppression and the potential implications

Continuous high frequency subthalamic DBS is an effective therapy for advanced Parkinson's disease, which raises the questions of: (1) whether aDBS, which requires simultaneous sensing and stimulation, is necessary, and (2) whether the consideration for efficacy and complexity balance still justifies the research on aDBS for PD. Continuous high frequency STN DBS induces changes in the network beyond the suppression of pathological synchrony in the beta frequency band. It functionally disconnects STN from the basal ganglia network.

Research from Dr. Huiling Tan's lab has shown that continuous high frequency STN DBS can reduce activities over a broad frequency band including alpha and gamma oscillations (Wiest et al., 2023b). Recent studies have also reported the high frequency resonant neural activity (ERNA) in the STN and the GPi (Sinclair et al., 2019; Johnson et al., 2023; Wiest et al., 2023a), and the amplitude and frequency of the ERNA change with time after the stimulation is switched on, reflecting synaptic depletion. These changes are not specific to Parkinson's disease, but also observed in cervical dystonia (Wiest et al., 2023b).

Continuous high frequency STN DBS over-suppresses gamma oscillations during voluntary movements, which helps invigorate the movements, compared to beta-triggered aDBS. Therefore, even though beta-triggered aDBS led to less stimulation during reaching movements, it improves the movement performance to the same level as continuous DBS (He et al., 2023). Meanwhile, the ERNA reduced in frequency and amplitude with time during cDBS indicating synaptic depletion, whereas ERNA remained high during aDBS due to the bursting nature of the DBS, indicating a different synaptic status (Wiest et al., 2023a).

The mechanism of continuous DBS also has an impact on sleep. Evidence has started to converge that beta bursts in STN LFPs persist during NREM sleep in human patients, although the occurrence is less frequent. Increased beta and low gamma during NREM correlated with arousal and sleep fragmentation index, which will be reduced by cDBS. However, cDBS may interrupt other physiologically important activities during sleep such as the sleep spindles, slow waves, and their temporal relationship. aDBS has the potential to suppress the pathological signals underlying sleep fragmentation, and at the same time preserve or increase those activities that are functionally important to recover normal sleep structure.

### Do the parkinsonian basal ganglia dream of beta oscillations?

Sleep has robust effects on discharge rate and pattern of neurons in the basal ganglia of naïve (normal, control) non-human primates (NHPs). NHPs treated with MPTP exhibit massive degeneration of midbrain dopaminergic neurons, depletion of striatal dopamine, as well as severe sleep fragmentation. Synchronous beta oscillations persist during rapid eye movement (REM) and non-REM (NREM) sleep and entrain the cortical EEG. LFP beta oscillations recorded from the basal ganglia of Parkinson's patients exhibit a robust circadian rhythm; however, even during NREM sleep the level of beta oscillations is higher than in the control (dystonia).

DBS techniques are improving. Classical DBS therapy is given continuously (24/7), probably inactivating the basal ganglia (by high-frequency depletion of afferent and efferent synaptic vesicles) and allowing compensation by the cortical networks. Current sensing devices can enable closed-loop DBS therapy that inactivate the basal ganglia only when they “misbehave” (i.e., during long episodes of beta oscillations). Future closed-loop DBS therapy would use phase, frequency, and circadian information to suppress beta oscillations and to augment sleep delta oscillations. This would restore normal sleep architecture and remove accumulated misfolded proteins and may slow down the progression of REM sleep behavior disorder and Parkinson's disease.

### Going on with DBS and gait

Parkinsonian gait disorder and freezing of gait (FoG) remain therapeutic challenges. Besides current troubleshooting options for STN DBS (Pötter-Nerger and Volkmann, 2013), further approaches should be based on hypotheses of gait pathophysiology. Recent intraoperative observations from microelectrode recordings of basal ganglia single neurons during a stepping task in PD revealed a nucleus specific modulation of single unit activity with nigral neurons (SN) modulated during attentional, cognitive cues (Gulberti et al., 2023), supporting the therapeutical approach of STN-SN co-stimulation. In line with these findings, combined STN+SN-DBS improved gait quality in postoperative patients particularly during gait conditions with increased attentional load (Horn et al., 2022). Another hypothesis proposes FoG to be a phenomenon of chronic “overstimulation” by DBS. One potential option would be a down-regulated, fiber-specific “short-pulse” stimulation, which was shown to equally improve objective gait parameters as conventional DBS in the short-term, but subjectively preferred in about 40% of freezing PD patients (Seger et al., 2021). Future hopes include potential DBS algorithms that drive beneficial neuronal plasticity (Horn et al., 2020) to normalize basal ganglia circuit activity and prevent or delay FoG onset in later stages of the disease. So far, the complex context of the parkinsonian gait disorder and methodological restraints limit the interpretation of DBS efficacy on gait.

## Advances in Asia

### Intracranial physiological biomarkers of neuropsychiatric symptoms

**Figure 5:**
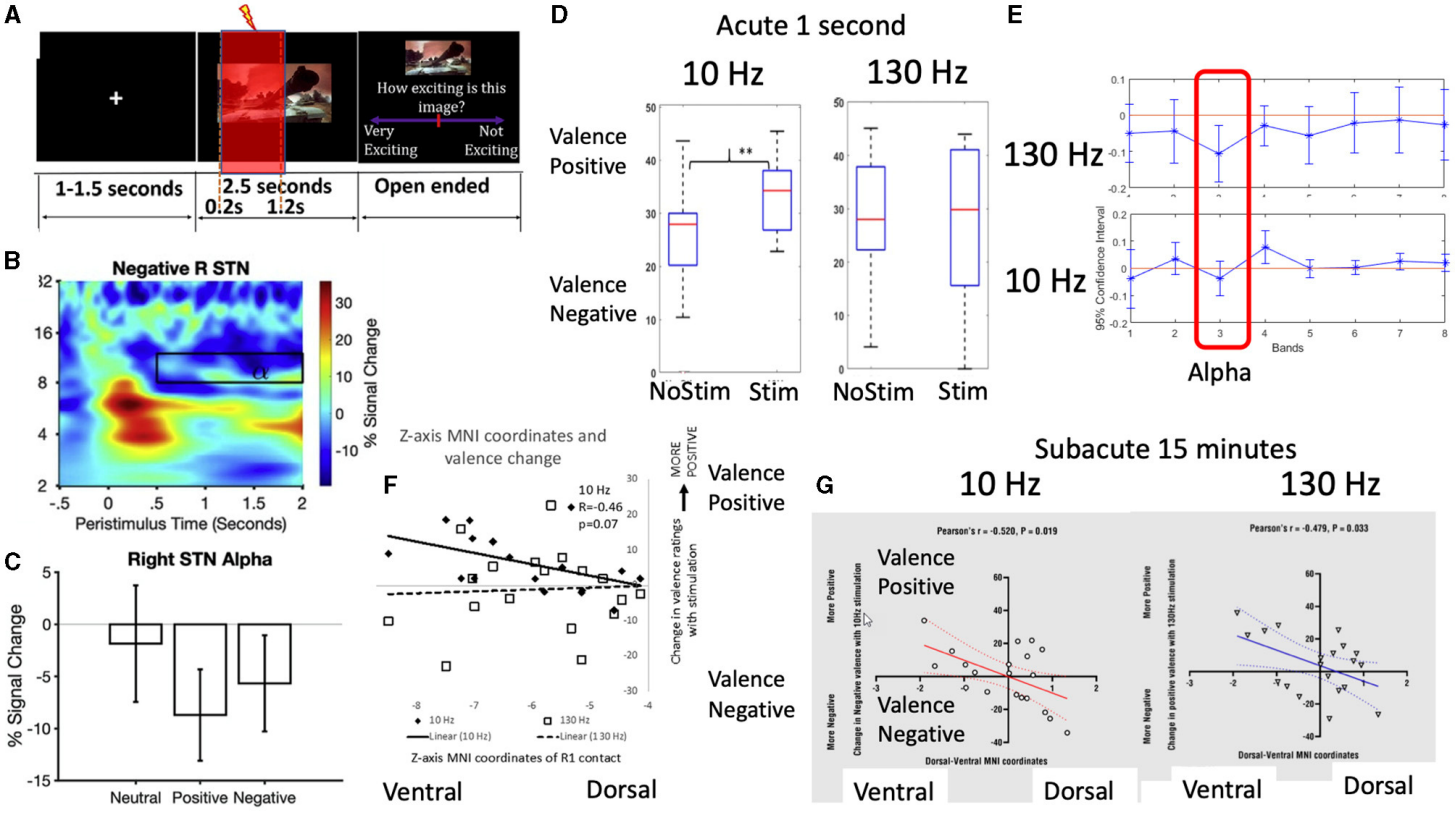
Subthalamic stimulation effects on emotional processing and alpha frequency. Intracranial subthalamic physiology recordings were paired with **(A)** a task-based emotional processing task with negative, positive and neutral images and recorded subjective valence and arousal (here shown with 1 second stimulation) (1). The results demonstrated the **(B, C)** expected late alpha desynchronization with affective imagery (1) which has been previously correlated with subjective valence and depression scores. **(D)** Using acute 1 second stimulation, there was a shift toward positive subjective valence bias to 10 Hz (alpha) stimulation but not 130 Hz (clinical) stimulation frequency (1) with **(E)** 10 Hz stimulation increasing alpha synchronization (i.e., loss of alpha desynchronization) (2). Both **(F)** acute 1 second 10 Hz stimulation and **(G)** subacute 15 min 10 Hz and 130 Hz stimulation of ventral contacts (3) was associated with greater positive emotional valence bias.

STN DBS can be associated with hypomania in both PD and OCD patients, particularly linked to ventral associative-limbic contacts. In the first set of studies, Dr. Valerie Voon's lab deconstructed hypomania operationalized into testable cognitive constructs. Using an emotional processing task, acute STN stimulation in PD patients at 10 Hz shifted to a positive subjective emotional bias (Mandali et al., 2020) and increased alpha power (Muhammad et al., 2023). This shift occurred with both acute (Mandali et al., 2020) and subacute stimulation with ventral contacts (Wang et al., 2023) (Figure 5). Second, high frequency acute STN stimulation of ventral contacts enhanced risk seeking in PD patients. Normative Lead-DBS and Biobank analyses dissociated risk-taking as a function of pre-supplementary motor area (SMA) and SMA anatomical and functional connectivity with STN. Thus, they highlight cognitive dimensions associated with ventral STN stimulation, representing key hypomania symptoms of euphoria and risk-seeking symptoms. In the final study, they highlight resting state theta activity in the bed nucleus of the stria terminalis, and prefrontal theta along with functional connectivity as a resting state biomarker predicting depression therapeutic improvement at 3 months in depressed patients.

### Dual target stimulation for treatment-refractory depression

DBS has emerged as a promising therapeutic approach for the treatment of patients with treatment-resistant depression (TRD). However, the underlying mechanisms through which DBS exerts its therapeutic effects are not yet fully elucidated. Previous studies have provided robust evidence indicating the crucial involvement of the bed nucleus of the stria terminalis (BNST) and the nucleus accumbens (NAc) neural circuitry in the processing of negative emotions. In this study, Dr. Bomin Sun and colleagues analyzed local field potentials (LFP) and explored functional connectivity in the circuit involving the BNST and the NAc to assess the mechanisms underlying the therapeutic effect of DBS.

This study recruited a total of 18 patients diagnosed with TRD. Synchronous implantation of intracranial DBS electrodes was performed in the BNST and NAc. LFPs were recorded using the implanted electrodes. Post-surgically, the participants' baseline symptoms were assessed, and LFP recordings were conducted. Following a 6-week duration of DBS treatment, reassessment and recording was performed. The patients were categorized into two groups based on the criterion of achieving a >50% improvement in the clinical scales. Differences in the LFP characteristics and functional connectivity of the BNST-NAc circuit were explored before and after DBS treatment, and between the two groups.

After 6 weeks of DBS treatment, the majority of subjects (13/18) showed significant improvements in depressive symptoms (>50% on Hamilton depression scale, which defined as response group). Compared to non-response group, response group showed significantly modulated oscillatory activities of gamma in BNST and theta in NAc, which had strong correlations with TRD symptoms. Also, the enhancement of unidirectional functional connectivity of the BNST-NAc circuit was exclusively observed, measured by Granger causality. Phase-amplitude coupling in the circuit also supported this functional connectivity, and its strength was also correlated with TRD symptoms. Finally, they found that BNST-NAc functional connectivity enhancement is specific to low-complexity time windows, which may be related to the modulatory effects of DBS.

Therapeutic DBS not only modulated the LFP activities at stimulation site, but also promoted the functional connectivity within the neural circuitry extending from discrete nuclei to complex neuronal networks. This suggests that local stimulation may affect downstream subcortical structures by modulating the output pattern of neuronal projections. The modulation of DBS-induced functional connectivity was specific to a transient time window of low complexity, suggesting that one of the ways in which DBS exerts its therapeutic effects may be to entrain disordered neuron populations, reducing background noise and integrating the BNST-NAc circuit.

### A variety of clinical responses to thalamic DBS in individual Tourette syndrome patients

**Figure 6:**
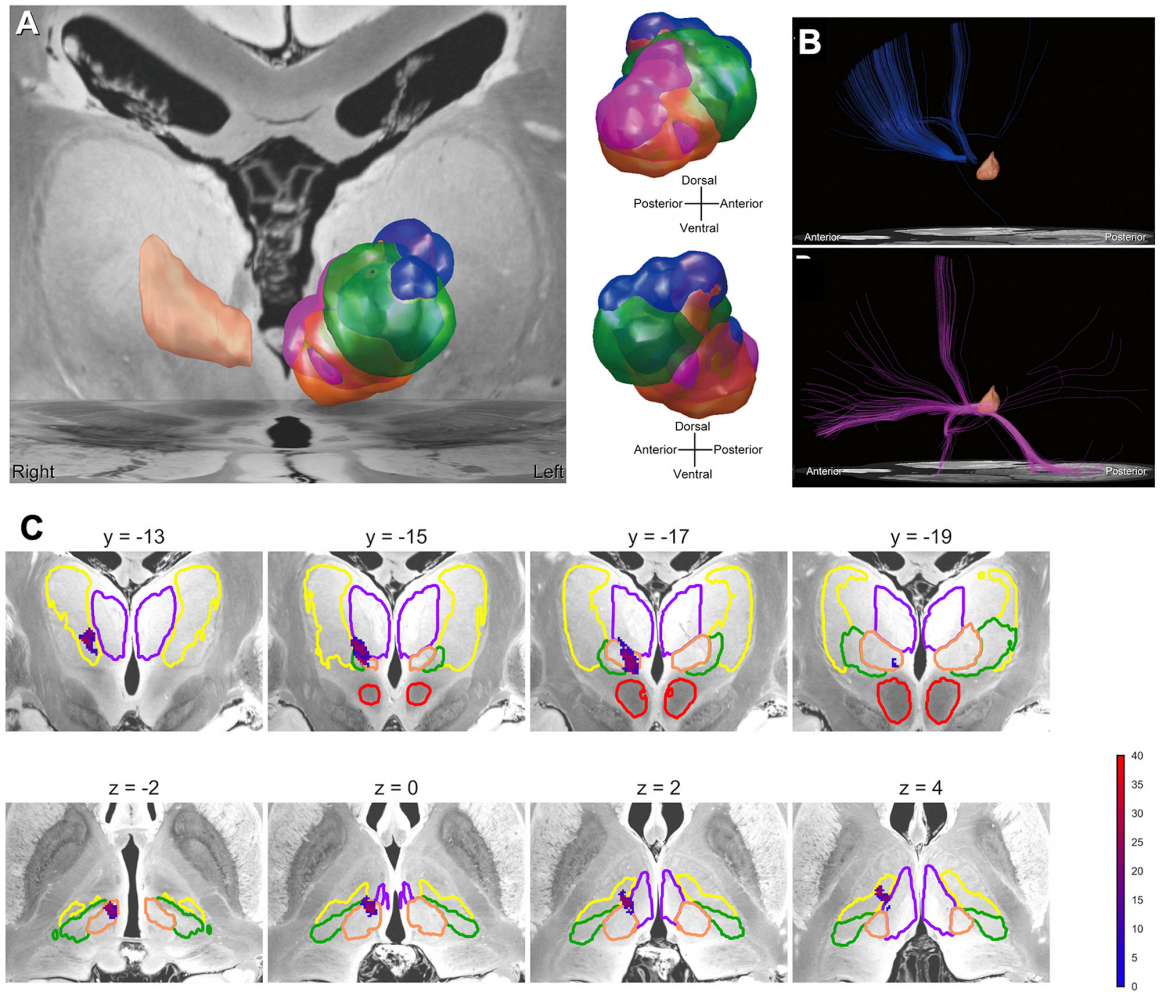
Mapping the effects of DBS for Tourette syndrome. **(A)** VTAs related to therapeutic stimulation and side effects. Each VTA color represents areas associated with the following effects: blue = therapeutic effect, orange = dizziness, green = paresthesia, and purple = depressed mood. The peach-colored region in the right hemisphere is the CM nucleus. **(B)** Normative connectome from VTAs related to therapeutic stimulation and side effects. The normative connectome from VTAs related to the therapeutic stimulation (above), and depressed mood (below). The peach-colored region is the CM nucleus. **(C)** Precision mapping of implanted deep brain stimulation electrodes in an atlas associated with tic improvements in the thalamus. The color bar represents the percentage improvement in tic symptoms.

The clinical responses to deep brain stimulation (DBS) for severe Tourette syndrome (TS) reportedly vary among patients. Possible solutions to achieve standardized outcomes may be precisely placing DBS electrodes in the “sweet spot” and selecting optimal stimulation parameters. Recent papers have addressed the position of a potential sweet spot and the importance of the neuroanatomy-based approach to DBS programming based on the microlesion effect of the centromedian (CM) thalamic DBS (Morishita et al., 2022) and connectomic analyses (Morishita et al., 2021) respectively (Figure 6). These reports have indicated that the dorsal border of anteromedial CM and ventrolateral thalamic nucleus may be a sweet spot. Another issue to be considered for successful TS is stimulation-induced neuroplasticity, as recent papers addressed that CM thalamic DBS may result in either beneficial or ominous effects in the long term (Smeets et al., 2017; Kimura et al., 2019). To induce beneficial neuroplasticity, selecting the optimal stimulation parameters is critical based on careful observations of the clinical symptoms. To evaluate the effects of CM thalamic DBS, computational measures as proposed for obsessive-compulsive disorder (Sakai et al., 2022) may be applied for TS, and this kind of computational modeling of behavior may be helpful to guide the optimal clinical course of TS patients who receive DBS therapy.

## Neuroethics cases: dilemmas that inform the future of neuromodulation

### Life after a beneficial experimental DBS trial: new opportunities and challenges

**Figure 7:**
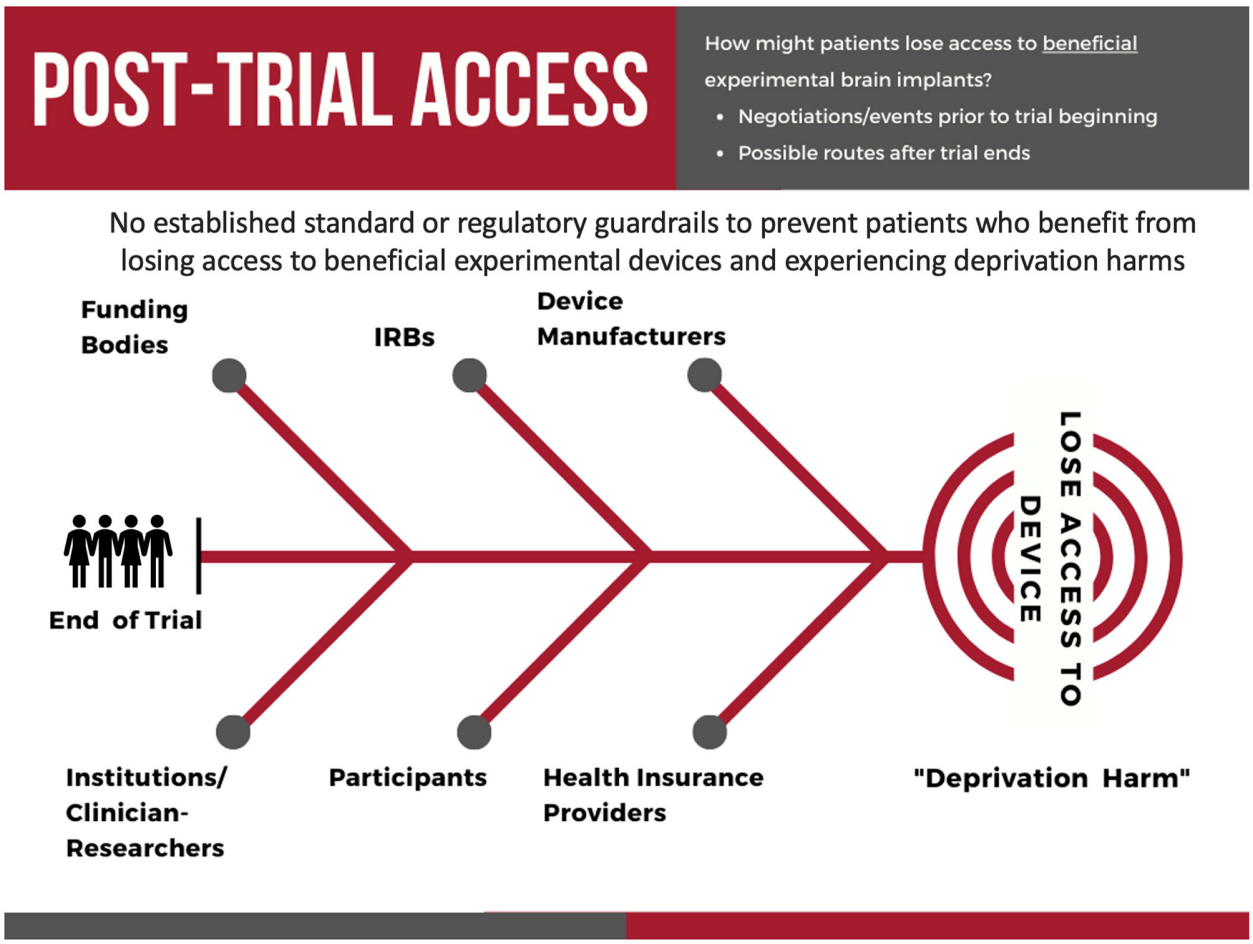
The key stakeholders involved in and potential pathways by which participants in experimental neural device trials may lose access to their neural implant after the conclusion of a clinical trial.

In the U.S. and other countries, patients with severe treatment-resistant conditions (e.g., refractory depression, OCD) whose symptoms improve with an implantable neural device during a clinical trial, may not be able to keep the device functioning after the trial ends (Underwood, 2017; Lázaro-Muñoz et al., 2018, 2022; Drew, 2020, 2022). There are several potential factors that can contribute to losing access to a device, including the Institutional Review Boards not requiring a plan for post-trial care for the study to be approved, funding bodies may not be willing or able to assist, or the research teams who conduct the trials may not be able to negotiate post-trial access with the device manufacturers (Figure 7).

In the U.S., even if a participant's treatment-resistant conditions improve with the experimental device, health insurance providers may deny coverage for post-trial device maintenance on the basis that the device is being used for an experimental indication in the trial and determine it is not “medically necessary” (Lázaro-Muñoz et al., 2018). This would shift the financial burden to participants, which can be prohibitively expensive for many individuals. Finally, even if the participants can finance the costs of post-trial care, device manufacturers may go out of business, cease production of the device or some necessary component of the device, or stop servicing the necessary software, leaving patients without access (Strickland and Harris, 2022). This can create unease among former trial participants and lead to deprivation harms, as a DBS trial participant stated: “*It would be kind of cruel to give a guy this much life back and snatch it away just because of a few pennies, although it's more than a few pennies*” (Lázaro-Muñoz et al., 2022; Hendriks et al., 2023). Implantable device deprivation harms (IDDH) risk exposing patients to severe rebound effects if the device stops functioning (Vora et al., 2012) and could lead to further morbidity or even mortality (e.g., DBS for TRD and increased risk for suicide).

Dr. Gabriel Lázaro-Muñoz and colleagues are calling for stakeholders, including funders, device developers, institutional review boards, hospitals/researchers, to develop adequate post-trial *maintenance* plans (PTMP) to ensure access to the necessary hardware, software, and maintenance for a period of time after the trial ends (e.g., 5 years of maintenance from the time the trial ends). This will allow participants further time to strengthen their case that health insurance providers should cover some costs, allow time for the device to be approved by the FDA, or allow the participant time to look for other alternatives. What we cannot allow as a society is to have people with severe treatment-resistant conditions, who have contributed to our scientific and medical understanding of the brain by allowing us to use their brains to test these devices, go back to life with a severe treatment-resistant condition when we have the technology to manage their condition already implanted in their bodies.

### Neurotech justice at the bedside: thinking about what we say to patients about technology

**Figure 8:**
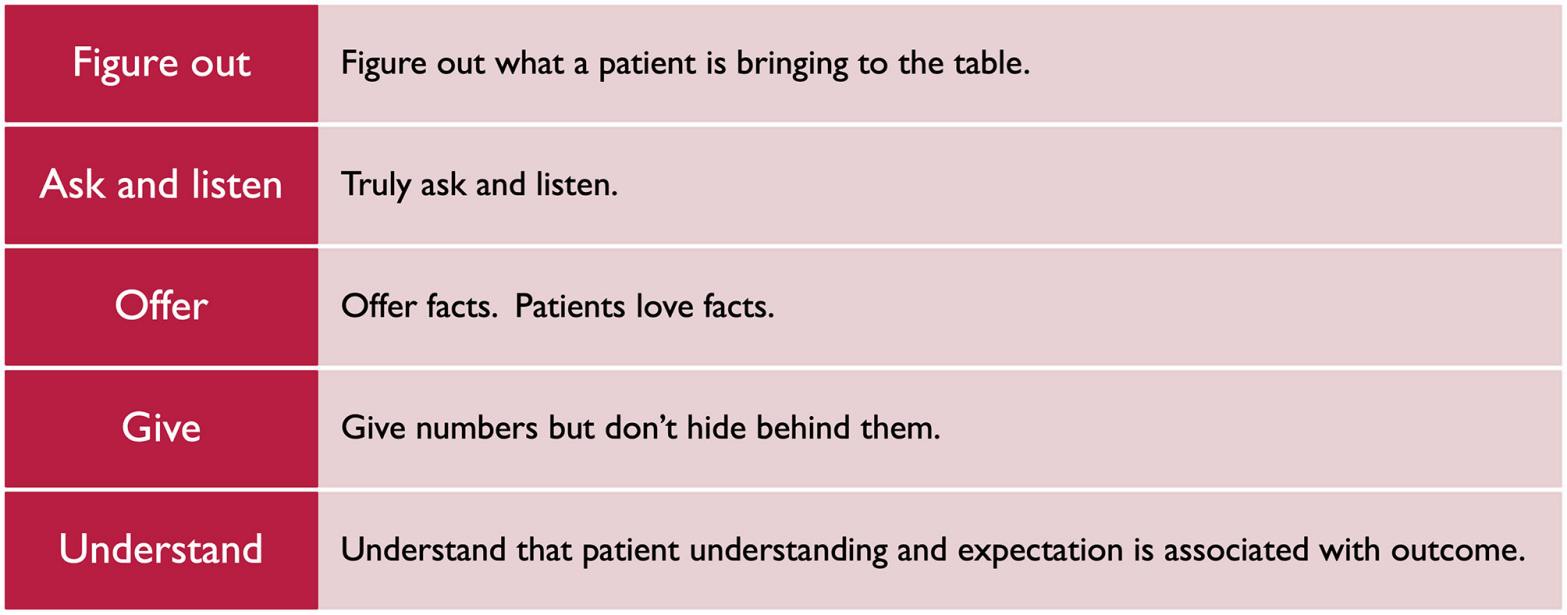
Framework for bidirectional, surgeon and patient communication.

Neuromodulation has a reputation problem. Referrers and patients alike view neurosurgical interventions as risky and often do not associate neurosurgical treatment with improved function or quality of life (Mitchell et al., 2023). Indeed, a recent study showed that of the 2,487,819 patients in the National Inpatient Sample with movement disorders between 2012 and 2019, only 29,820 had a procedure involving neuromodulation. This number was markedly less for patients of color as well as rural patients, indicating a disparity in access to care. This access disparity likely represents a multifactorial problem from sociodemographic barriers, to care to patient and provider perceptions of surgery, to provider and systemic biases. Dr. Theresa Williamson's studies have shown that Black and Hispanic patients for example report worse quality of life outcomes and pain and physical dysfunction while also being less likely to undergo surgical treatment [(Williamson, 2023) and in press]. One proposed mechanism for bias and potential for improvement in neuromodulation equity is to improve a patient's understanding of surgical benefit and risk and trust in a provider by tailoring our communication with patients who have surgical pathology. Their studies show that all patients and patients of color find shared decision-making pamphlets and conversations to be easy to use, important, and meaningful. Figure 8 illustrates key to communication to improve goal-concordant care.

### Keeping the human in the loop: considerations regarding trust, responsibility, and justice

Large investments to address the failure of, or provide an alternative to, pharmacotherapy and other interventional strategies for neurologic disease and major mental health disorders have led to significant advances in DBS, including the introduction of rechargeable systems, directional electrodes, and adaptive systems. However, as we develop and use these advances it is key to *keep the human in the loop*. People's attitudes and beliefs about these technologies are key to their acceptance and uptake, and addressing structural and attitudinal barriers is key to improve their availability and accessibility. Dr. Laura Cabrera provided three considerations as we think on the importance of keeping the human in the loop. The first relates to social justice considerations. No matter how good the technology developed is, it is problematic if people that need it cannot get it; this includes issues of geographic availability of centers offering these technologies to lack of physicians willing to refer and continue caring for patients implanted. A second practical consideration relates to trust – not only trust toward the medical system, but also the patient's trust toward their devices. Finally, the use of these types of devices might put into question views about who or what is responsible for outcomes.

## Artificial intelligence in neuromodulation

### Clinical decision support systems for DBS

The field of clinical DBS is currently benefiting from amazing new technological developments in both the hardware and software tools associated with commercial DBS systems. However, the financial investments and industrial decisions that enabled those new clinical technologies were made 10+ years ago, during a time of lofty expectations for clinical and commercial growth in DBS. Unfortunately, those predictions have proven inaccurate, with anemic growth over last few years. The exact root of this issue is certainly multifactorial. Nonetheless, it is clear that something needs to change to reignite clinical growth if we are to justify the continuation of large R&D budget allocations to DBS. One hypothesis is that the field of clinical DBS has reached a bandwidth ceiling for working within a tertiary care center model. As such, an opportunity to facilitate growth may be in democratizing the management and analysis of DBS patients to a wider spectrum of neurologists via algorithmic tools. In turn, academic and industrial research teams have been working to develop prototype systems for many years, and the first wave of FDA-approved AI-based DBS clinical decision support systems are becoming available.

### If Netflix did neuromodulation: a framework for behaviorally driven, scalable programming

**Figure 9:**
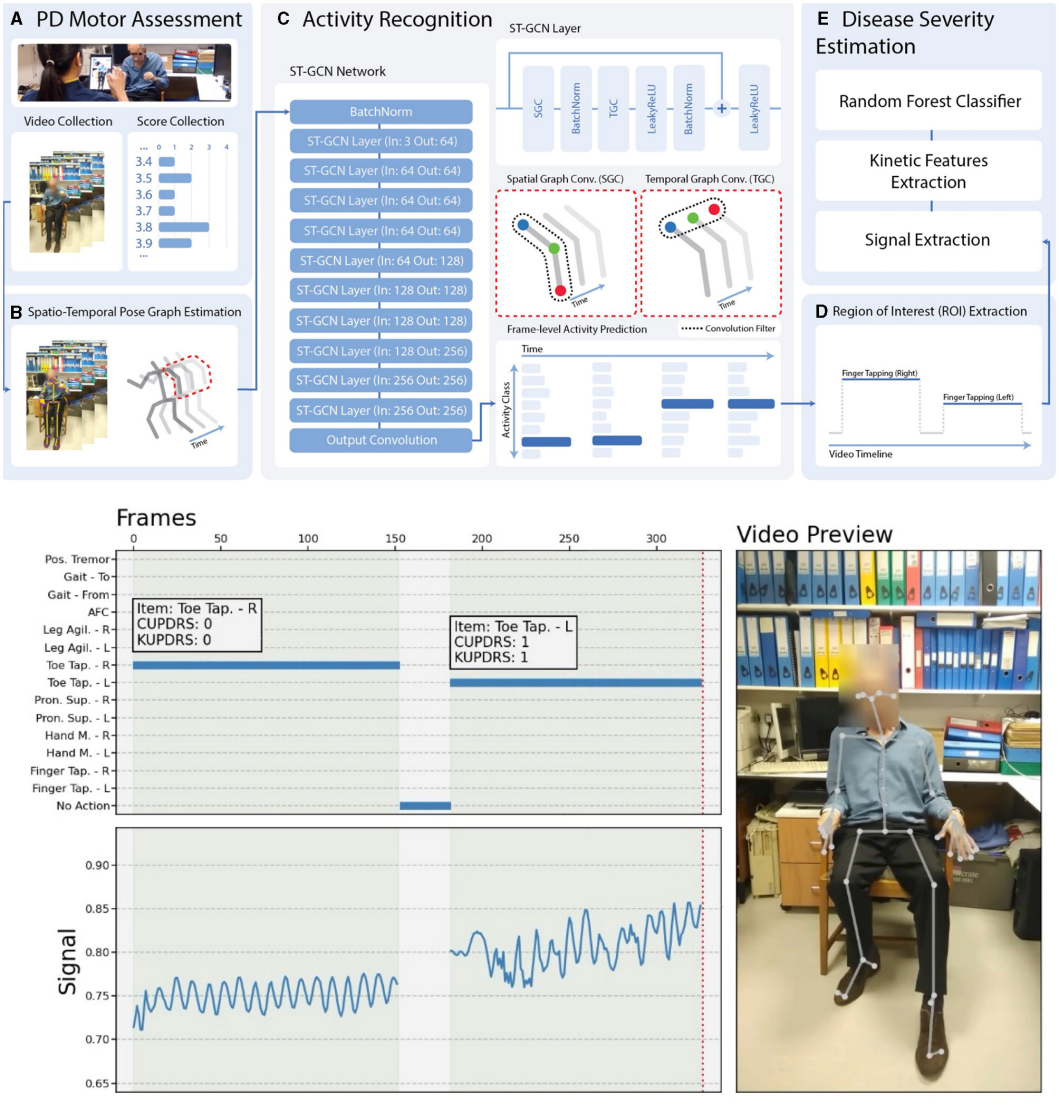
Fully automated, video-based motor assessment in Parkinson's disease. Upper half: **(A–E)** Stages of processing a patient video passes through to arrive at a disease severity rating. Lower half: The right panel shows an example video once processed. The upper left panel shows the output of activity recognition (*right toe-tapping*, followed by *left toe-tapping*, with *no activity* indicated in intervening periods). The white boxes show the clinical label (CUPDRS) and the model output (KUPDRS). The lower left panel shows the clinical signal extracted during a relevant activity (in this case, toe-tapping), the features of which are passed to a model for inference.

Assessment of the human motor system is a core component of evaluating and monitoring neurological diseases. However, today it is typically confined to the clinic. Machine Medicine collected a large dataset of high-quality, clinician-labeled clinical videos of MDS UPDRS-III motor assessments. Applying machine learning and software engineering across the stack (i.e., mobile, web, DevOps, and algorithm training and design), they developed and launched a fully automated pipeline, including administration of an abbreviated motor assessment, activity recognition, and disease severity estimation (Figure 9). Data capture requires the patient's engagement and their smartphone or tablet; otherwise, the pipeline requires no input from a clinician or other human agent, meaning it is highly scalable. This tool could be applied to data-intensive problems such as programming numerous neuromodulation devices without significantly burdening the clinician, while also delivering an uncomplicated patient experience.

Finally, the technical components required for such a scalable patient-centric system exist in the ecosystem today and, if combined correctly, could employ approaches pioneered by entities such as Amazon and Netflix in their recommender engines to solve conceptually similar and computationally comparable problems at scale.

### Empowering precision medicine: AI-driven medical imaging for personalized clinical applications

AI has revolutionized medical imaging, particularly in the context of DBS procedures. In recent years, there has been an exponential growth in studies using AI for medical imaging across three main categories: (1) Image analysis and segmentation, (2) image reconstruction, and more recently, (3) artifact correction and denoising methods. These capabilities are driven by several factors, including the maturation of deep learning algorithms, increased localized computing power, accessible labeled datasets, and the growing awareness of the potential benefits of deep learning in medical imaging.

Focusing on DBS surgery, the impact of AI is profound. A fully automated segmentation process offers several clear advantages, including accuracy (unbiased) and speed (seconds) of inference. It enables improved target localization precision by accounting for patient-specific anatomical differences and reducing electrode placement variability, thereby leading to better outcomes.

AI's impact on medical imaging extends to expanded access to care. AI technology could alleviate the scarcity of specialized neurosurgeons by enabling less experienced practitioners to perform DBS procedures. It also enables tailoring of treatment plans to individual patients.

Although significant advances have been made, there are still challenges to overcome in achieving optimal accuracy and precision. These challenges are related to the quality and diversity of training data, which can vary in terms of imaging protocols, data quality, and anatomical differences. Overall, AI's transformative potential in medical imaging for DBS surgery promises enhanced accuracy, safety, accessibility, and efficiency.

## Time scale to measure signatures of mood abnormalities

Treatment-resistant depression (TRD) is a complex neuropsychiatric syndrome that is highly heterogeneous across individuals, and symptoms often fluctuate over many time scales. Increasing evidence suggests DBS can be effective to treat select cases of TRD, but the time scale at which symptoms and biomarkers of TRD should be measured when evaluating the effects of DBS is not well established. Two experts in DBS for TRD provided their rationale and supporting evidence for different time scales.

### Days to weeks is both adequate and doable with SCC DBS

Continuous high frequency subcallosal cingulate (SCC) stimulation is one of various strategies for delivering DBS therapy for treatment-resistant depression (TRD) (Mayberg et al., 2005; Holtzheimer et al., 2012). Sustained antidepressant effects can be maintained over many years with SCC DBS using standardized stimulation parameters and minimum current adjustments (Kennedy et al., 2011; Crowell et al., 2019). Leveraging this clinical experience, a standardized method for lead implantation has been developed and validated using patient-specific tractography (Riva-Posse et al., 2014) and reliably executed with consistent outcomes across two centers and four cohorts (Mayberg et al., 2023). However, it remains difficult even for clinical experts to discriminate nonspecific or transient mood symptoms from persistent symptoms that might benefit from dose adjustments. Even when adjustments are made, it takes several weeks to see meaningful clinical effects. Therefore, brain biomarkers that could indicate when a stable response has been achieved and that could also inform when an adjustment is required would have obvious clinical utility. The availability of prototype sensing systems (Medtronic, ACTIVA PC+S; SUMMIT RC+S), embedded in conventional DBS devices, provided a new platform to develop such a readout as part of established clinical protocols (Alagapan et al., 2023). With a focus on past observations of stereotypic acute effects (Choi et al., 2015; Sendi et al., 2021) and the predictable evolution of antidepressant effects over weeks to months across patients, LFP patterns from the SCC were first interrogated to define a common biomarker of the desired outcome—a stable antidepressant response. A neural net classifier was first used to differentiate LFP spectral features corresponding to the beginning and end of six-months of DBS therapy. Next, a novel tool, generative causal explainer (GCE), was used to identify discriminative factors that explained the classifier performance. A separate classifier could be similarly used to differentiate facial features extracted from videos of clinical interviews at the same time points. Coming full circle, the variance in patient response trajectories was correlated with the degree of white matter damage to the DBS target networked defined using DTI; patients with more severe abnormalities did not require a separate classifier, but rather just more time to achieve stable recovery (Alagapan et al., 2023). These first findings appear to replicate in a new cohort of patients (Mayberg et al., 2023). While a control policy has not yet been implemented, these findings support the argument that days to weeks is both an adequate and clinically meaningful time scale to measure and treat a neural signature of major depression across individuals.

### Acute effects of patient-specific biomarkers to guide DBS

Dr. Andrew Krystal and colleagues are in the process of carrying out a study of intracranial closed-loop DBS. In this study, stimulus location and parameters are optimized for each patient during a 10-day inpatient stimulus-response mapping period, which is designed to address two of these unresolved issues by testing two fundamental hypotheses regarding DBS for TRD: (1) It is possible to elicit a repeatable immediate response, which then enables stimulus optimization/personalization, and (2) It is possible to achieve sustained therapeutic effects without stimulating continuously by implementing a closed-loop DBS paradigm where stimulus is triggered by an iEEG biomarker of depression severity. They have so far enrolled 4 subjects in the study and the results indicate that: (1) immediate therapeutic antidepressant effects can be reliably and repeatedly elicited with stimulation for at least one site/parameter set; and (2) DBS when delivered intermittently as part of a closed-loop paradigm where stimulus is delivered driven by a personalized iEEG biomarker of depression severity can lead to sustained antidepressant effects. These findings support the argument that acute neural signatures of TRD can be used to drive DBS.

## Features essential for future DBS devices

Despite some technological innovations, current neurostimulators are essentially unchanged since the inception of contemporary DBS, still employing open-loop fixed-frequency constant stimulation. Yet insights into mechanisms and effects of therapeutic stimulation, a greater understanding of disease pathophysiology, and advances in engineering have raised the prospect of delivering truly novel neurostimulators in the near future (Guidetti et al., 2021).

### Brain sensing

Brain sensing is amongst the most important features to include in neurostimulators, both because of the value of sensing and the prospect of providing dynamic, responsive therapy. The rationale includes: (1) Pathophysiology of disease: disease, symptoms, and disease-related oscillatory changes are dynamic, making dynamic monitoring valuable (O'Keeffe et al., 2020; Xiao et al., 2023); (2) Therapeutic mechanisms of DBS: DBS modulates brain oscillations, making brain sensing a sensible means of monitoring therapy dosing and efficacy (Arlotti et al., 2018); (3) Need for biomarker-guided therapy: while DBS for movement disorders has an immediate measurable therapeutic response, emerging DBS indications (e.g., depression) require biomarkers to judge therapeutic efficacy on a timescale relevant for programming; (4) Improved efficacy: evidence suggests aDBS may be superior to contemporary DBS (Little et al., 2013; Swann et al., 2018; Cagle et al., 2022); (5) Safety: responsive neurostimulation offers the prospect of reducing stimulation and thereby reducing off-target side effects (Little et al., 2016; Rosa et al., 2017); and (6) Chronic/remote monitoring: sensing can provide chronic and remote sensing of therapy and patient state, to enhance overall patient care and therapy. Beyond brain sensing, the field may ultimately benefit from integrating other sensors as well, including both physiological [e.g., autonomic, heart rate (Huang et al., 2022)] and behavioral measures (e.g., activity).

### Alternative stimulation patterns

Almost all clinical use of DBS has involved the delivery of biphasic square wave stimulation at a constant frequency and a constant interpulse interval. The capability to deliver bursts of stimulation pulses at customizable frequencies and customizable interpulse intervals would help the field leverage promising findings from animal work and early human work. Optogenetic experiments have identified stimulation patterns that include variable interpulse interval, as capable of providing more cell type selective stimulation, or better treatment of specific motor signs in movement disorders (Spix et al., 2021). Theta burst stimulation may be more plasticity-inducing than standard constant frequency stimulation. Several human clinical trials of theta burst stimulation have suggested benefit in individual cases (Horn et al., 2020; Sáenz-Farret et al., 2021), but long term use of theta burst stimulation has not been investigated. Coordinated reset stimulation was introduced in animal models and has the potential to provide long lasting effects in the absence of ongoing stimulation (Tass, 2003; Tass et al., 2012). However, coordinated reset stimulation has only been tested in one human trial with externalized leads (Adamchic et al., 2014). Delivery of individual pulses time locked to the phase of a field potential oscillation could selectively reduce or enhance particular frequencies for therapeutic benefit (Cagnan et al., 2017). The lack of evidence in humans for efficacy of alternative stimulation patterns may be related to the limited capability of contemporary commercial DBS devices to deliver variable patterns.

### Neuroimaging and DBS fiber-filtering

**Figure 10:**
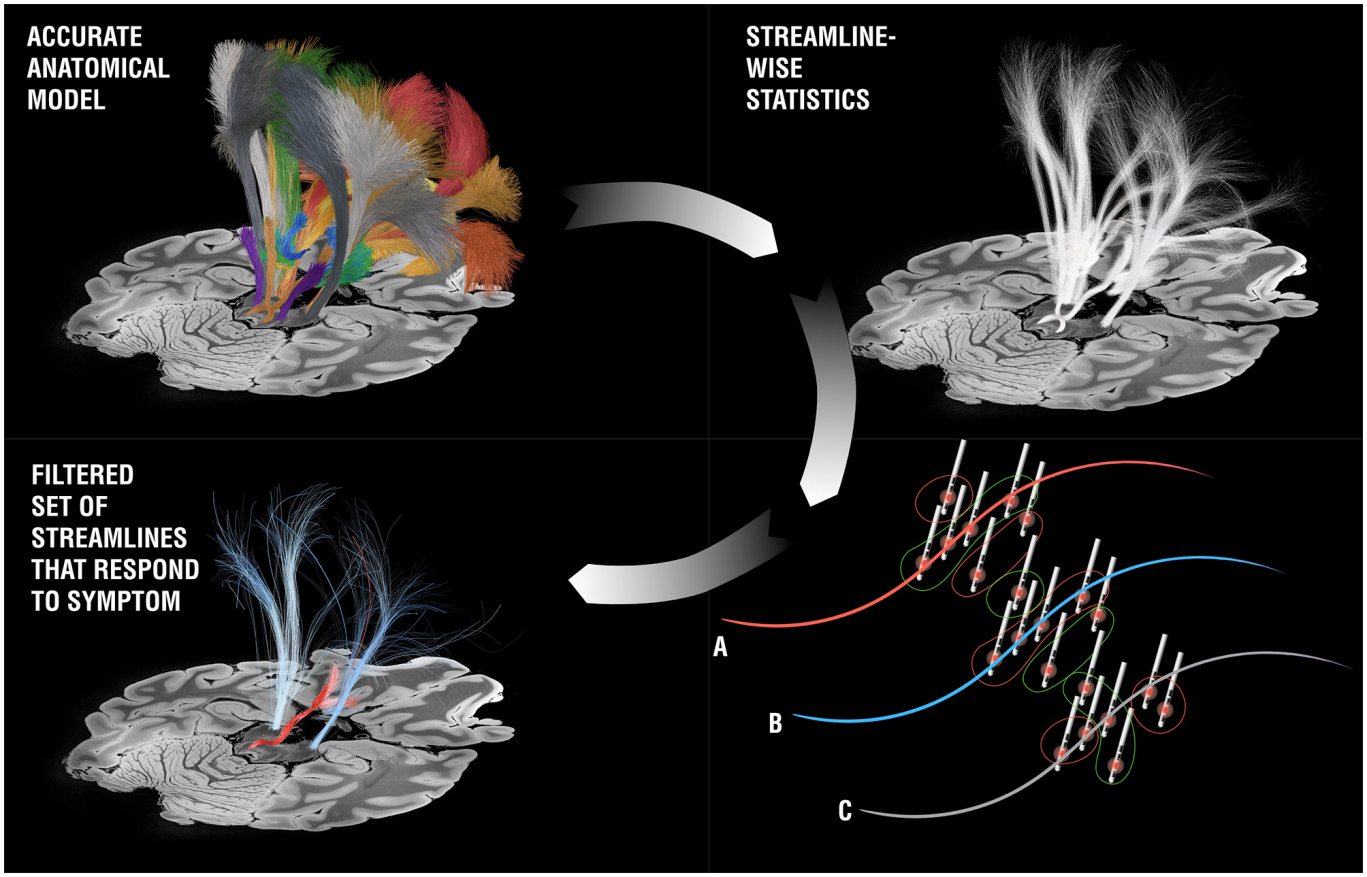
DBS fiber-filtering. Based on an anatomical model [pathway atlas or normative connectome, the basal ganglia pathway atlas (Petersen et al., 2019) is shown as an example, top panels], each streamline is selected and set into relationship with DBS stimulation volumes from a cohort of patients in a mass-univariate statistical approach. For each streamline, three general scenarios may result: **(A)** The streamline was predominantly modulated in patients in which a symptom of question was reduced, but less in patients in which the symptom deteriorated or stayed stable. If this is the case, a positive score is assigned to the streamline (these streamlines have been visualized with red color in most publications). **(B)** The opposite case: The streamline was predominantly modulated in the patients in which a symptom got worse (the tract receives a negative score and is often shown in blue). **(C)** There is no clear relationship between modulation of the streamline and changes of the symptom of question. In this case, the streamline is filtered out. When iteratively applied across all streamlines in the atlas, the bundles coding for improvements of a specific symptom can be identified (here the cerebellothalamic pathway for tremor improvement in right-lateralized DBS electrodes).

One prominent avenue to personalize DBS surgery and programming that has been proposed lies in gathering individual high quality MRI datasets that include diffusion-weighted imaging scans for tractography or specialized MRI sequences that allow for a better visualization of the DBS target (Neudorfer et al., 2022). An alternative strategy was recently proposed that involves three consecutive steps (Hollunder et al., 2021). The first involves identifying networks that, when stimulated, respond by modulating specific symptoms (such as tremor, bradykinesia, rigidity, or axial symptoms in the case of Parkinson's disease). Notably, and slightly counter-intuitively, these networks would be identified on a group level in a normative atlas space, e.g. using DBS network mapping (Horn et al., 2017) or DBS fiber filtering [Figure 10; (Baldermann et al., 2019; Li et al., 2020)]. In a second step, the normative networks could be reidentified in the individual patient by using individual tractography data, a process referred to as “template-matching” (Hollunder et al., 2021). The last step involves weighting the networks in a fashion that personalizes to the individual patient, termed “network-blending”. For instance, in a patient with predominant tremor, the tremor network would receive a strong weight and would be considered more strongly than the networks that respond to other symptoms. The resulting weighted blend of networks may define (i) a programming target for existing leads that have already been implanted, or, after successful establishment of the approach, (ii) a personalized and refined surgical target within established DBS target nuclei.

## Conclusion

The 11th Annual DBS Think Tank meeting facilitated productive discussions surrounding advances in neuromodulation research and the latest commercially available technologies. Translational research performed in animal models with optogenetics and VNS and in humans with functional neuroimaging and neurophysiology shows promise to shed light on the potential mechanisms underlying DBS in a variety of disorders. Additionally, neurophysiological markers are increasingly being evaluated as control signals for aDBS as the next-generation paradigm to improve efficiency and efficacy. Tailoring aDBS algorithms to account for sleep and circadian patterns was a key point of discussion. Current commercially available technologies feature chronic sensing, imaging- and outcomes-guided DBS programming algorithms, digital health integration and cloud-based management systems, responsive stimulation for emerging epilepsy indications, the use of biosensors to guide the treatment of Parkinson's disease, and a commercial aDBS device. Additionally, many discussions focused on developing and evaluating neurophysiological and imaging markers to guide DBS for neuropsychiatric indications, such as OCD, TS, and depression. Attendees expressed unanimous support for an aggressive immediate solution for providing enhanced access to DBS devices to smaller and more rare indications. Despite many technological and research advancements, the field of DBS is at a crossroads, and the use of AI and data-driven approaches will likely be key to advance DBS as a widespread therapy.

## Data availability statement

The original contributions presented in the study are included in the article/supplementary material. Further inquiries can be directed to the corresponding author.

## Ethics statement

The studies involving humans were approved by the Individual Academic Institutions. The studies were conducted in accordance with the local legislation and institutional requirements. The participants provided their written informed consent to participate in the studies. Animal studies were approved by individual institutional animal welfare committees at the respective academic institutions. All studies were conducted in accordance with the local legislation and institutional requirements.

## Author contributions

KJ: Writing – original draft, Writing – review & editing. ND: Writing – original draft, Writing – review & editing. EG: Writing – original draft, Writing – review & editing. CW: Writing – original draft, Writing – review & editing. KW: Writing – original draft, Writing – review & editing. HB-S: Writing – original draft, Writing – review & editing. VV: Writing – original draft, Writing – review & editing. TM: Writing – original draft, Writing – review & editing. YS: Writing – original draft, Writing – review & editing. AM: Writing – original draft, Writing – review & editing. GL-M: Writing – original draft, Writing – review & editing. TW: Writing – original draft, Writing – review & editing. AH: Writing – original draft, Writing – review & editing. RG: Writing – original draft, Writing – review & editing. JO'K: Writing – original draft, Writing – review & editing. AG: Writing – original draft, Writing – review & editing. W-JN: Writing – original draft, Writing – review & editing. SL: Writing – original draft, Writing – review & editing. NP: Writing – original draft, Writing – review & editing. SS: Writing – original draft, Writing – review & editing. AF: Writing – original draft, Writing – review & editing. AH-B: Writing – original draft, Writing – review & editing. RR: Writing – original draft, Writing – review & editing. LM: Writing – original draft, Writing – review & editing. YP: Writing – original draft, Writing – review & editing. DG: Writing – original draft, Writing – review & editing. SM: Writing – original draft, Writing – review & editing. LK: Writing – original draft, Writing – review & editing. HT: Writing – original draft, Writing – review & editing. HB: Writing – original draft, Writing – review & editing. MP-N: Writing – original draft, Writing – review & editing. BS: Writing – original draft, Writing – review & editing. LC: Writing – original draft, Writing – review & editing. CM: Writing – original draft, Writing – review & editing. NH: Writing – original draft, Writing – review & editing. HM: Writing – original draft, Writing – review & editing. AK: Writing – original draft, Writing – review & editing. NP: Writing – original draft, Writing – review & editing. PS: Writing – original draft, Writing – review & editing. KF: Writing – review & editing. MO: Writing – review & editing. JW: Writing – review & editing.
